# LIN-44/Wnt Directs Dendrite Outgrowth through LIN-17/Frizzled in *C. elegans* Neurons

**DOI:** 10.1371/journal.pbio.1001157

**Published:** 2011-09-20

**Authors:** Leonie Kirszenblat, Divya Pattabiraman, Massimo A. Hilliard

**Affiliations:** Queensland Brain Institute, The University of Queensland, Brisbane, Australia; University of Cambridge, United Kingdom

## Abstract

Nervous system function requires proper development of two functional and morphological domains of neurons, axons and dendrites. Although both these domains are equally important for signal transmission, our understanding of dendrite development remains relatively poor. Here, we show that in *C. elegans* the Wnt ligand, LIN-44, and its Frizzled receptor, LIN-17, regulate dendrite development of the PQR oxygen sensory neuron. In *lin-44* and *lin-17* mutants, PQR dendrites fail to form, display stunted growth, or are misrouted. Manipulation of temporal and spatial expression of LIN-44, combined with cell-ablation experiments, indicates that this molecule is patterned during embryogenesis and acts as an attractive cue to define the site from which the dendrite emerges. Genetic interaction between *lin-44* and *lin-17* suggests that the LIN-44 signal is transmitted through the LIN-17 receptor, which acts cell autonomously in PQR. Furthermore, we provide evidence that LIN-17 interacts with another Wnt molecule, EGL-20, and functions in parallel to MIG-1/Frizzled in this process. Taken together, our results reveal a crucial role for Wnt and Frizzled molecules in regulating dendrite development in vivo.

## Author Summary

Neurons have distinct compartments, which include axons and dendrites. Both of these compartments are essential for communication between neurons, as signals are received by dendrites and transmitted by axons. Although dendrites are vital for neural connectivity, very little is known about how they are formed. Here, we have investigated how dendrites develop in vivo by examining an oxygen sensory neuron (PQR) in the nematode *C. elegans*. Using a genetic approach, we have discovered that Wnt proteins, a group of highly conserved secreted morphogens, interact with their canonical Frizzled receptors to control the development of the PQR dendrite. We show that Wnt molecules act as attractive signals to determine the initiation and direction of dendrite outgrowth. Interestingly, Wnt proteins act specifically on the dendrite without affecting the axon, suggesting that outgrowth of the dendrite can be regulated by distinct processes that are independent of axon formation. We predict that similar mechanisms may be in place in other species owing to the conserved roles of Wnt and Frizzled molecules in development.

## Introduction

Correct dendrite development is essential for the establishment of neuronal connectivity and, in sensory neurons, for the detection of external stimuli. However, the complexity and variety in morphology of dendrites has made the study of their development more challenging than that of axons. Previous findings have shown that some axon guidance molecules can also regulate dendrite development, often with opposing effects. For example, the guidance cue Slit can simultaneously repel axons and enhance dendrite branching and outgrowth in cortical neurons [1]. Similarly, Semaphorin 3A, a guidance molecule that acts through the Neuropilin-1 receptor, functions as both a chemorepellent for cortical axons and a chemoattractant for dendrites within the same neurons [2]. The differential response of axons and dendrites to Semaphorin 3A is mediated by asymmetric localization of a soluble guanylate cyclase to the dendrites [2]. In cultured hippocampal neurons, local elevation of cAMP and reduction of cGMP in undifferentiated neurites promotes axon formation and suppresses dendrite formation, whereas the reciprocal levels of these molecules have the opposite effects [3]. Interestingly, local upregulation of cAMP in a single neurite results in long-range inhibition of cAMP levels in all other neurites, suggesting a mechanism for the development of one axon and multiple dendrites and indicating that dendrite formation in this context is secondary to axon formation [3].

More recently, in vivo studies have uncovered molecules that regulate dendrite development independently of the axon. Sensory neurons in the head of *C. elegans* develop by anchoring their dendritic tips to the nose while the cell body migrates away, extending a dendrite (retrograde extension) [4]. In the *C. elegans* tail motor neuron, DA9, the extracellular guidance cue UNC-6/Netrin controls the final extension of the dendrite in an axon-independent manner through its interaction with the receptor UNC-40/DCC [5]. In a different highly branched mechanosensory neuron, PVD, the cell-autonomous activity of the EFF-1 fusogen promotes branch retraction to retain a precise patterning of arbors during dendrite development [6]. In a *Drosophila* sensory neuron (vch'1), correct orientation of the dendrite is regulated by Netrin-A and its receptor Frazzled and is mediated by a migrating cap cell, which drags the tip of the dendrite into position [7]. In all these cases, however, the cell-intrinsic molecules involved in the initial stages of dendrite formation remain elusive.

Wnt morphogens and their Frizzled receptors are highly conserved molecules with diverse functions in nervous system development [8],[9]. In rat and mouse hippocampal neurons, Wnt molecules promote dendritic arborization [10],[11], whereas in *Drosophila* neuronal activity regulates the remodeling of dendritic branches in a Wnt-dependent manner [12]. In *C. elegans* there are five Wnt ligands, (LIN-44, EGL-20, CWN-1, CWN-2, and MOM-2) and four Frizzled receptors (LIN-17, MIG-1, CFZ-2, and MOM-5). The posteriorly expressed Wnt ligand, LIN-44, regulates neuronal polarity, axon guidance, axon termination, and synapse formation, acting mainly as a repellent through the LIN-17/Frizzled receptor on neurons in the posterior of the animal [13]–[17]. Another posteriorly expressed Wnt ligand, EGL-20, controls cell migration and axon guidance of different cells along the anterior-posterior axis of the worm [18]–[20]. CWN-1 and CWN-2, which are expressed more broadly along the anterior-posterior axis, affect neurons in the mid-body and the head of *C. elegans*, regulating neuron migration, axon guidance, nerve-ring placement, as well as the outgrowth and pruning of neurites [21]–[25].

In this study, we show that LIN-44/Wnt initiates and guides the development of the dendrite in the PQR oxygen sensory neuron, through a mechanism that occurs prior to and independently of the formation of the axon. In contrast to its role as a repellent in synapse formation and axon termination, in the context of PQR development LIN-44 acts as an attractant that is specific for the outgrowth of the dendrite. The effect of LIN-44 is mediated through the LIN-17 receptor, which functions in a cell-autonomous manner. We also identify EGL-20/Wnt and MIG-1/Frizzled as crucial molecules in PQR dendrite development. Taken together, these findings show for the first time that Wnt signals and Frizzled receptors can promote dendrite-specific outgrowth in developing neurons in vivo.

## Results

### Characterization of PQR Dendrite Development

**Figure 1:**
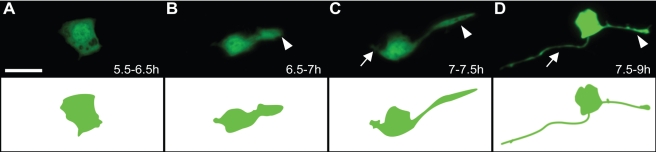
PQR development in wild-type *C. elegans*. PQR was visualized with *Pgcy-36::GFP*. (A–D) Normal development of the PQR neuron in wild-type larvae grown at 22°C. Hours post-hatching are indicated in each panel. (A) 5.5–6.5 h; PQR neuroblast prior to neurite development. (B) 6.5–7 h; the dendrite begins to form on the dorsal/posterior side of PQR and is visible as lamellipodial outgrowth (arrowhead). (C) 7–7.5 h; the dendrite extends posteriorly with a growth cone (arrowhead) and the axon emerges ventrally (arrow). (D) 7.5–9 h; the dendrite and axon have fully emerged and continue to extend. Anterior is to the left and ventral is down in these and all subsequent images. Scale bar: 5 µm.

**Figure 2:**
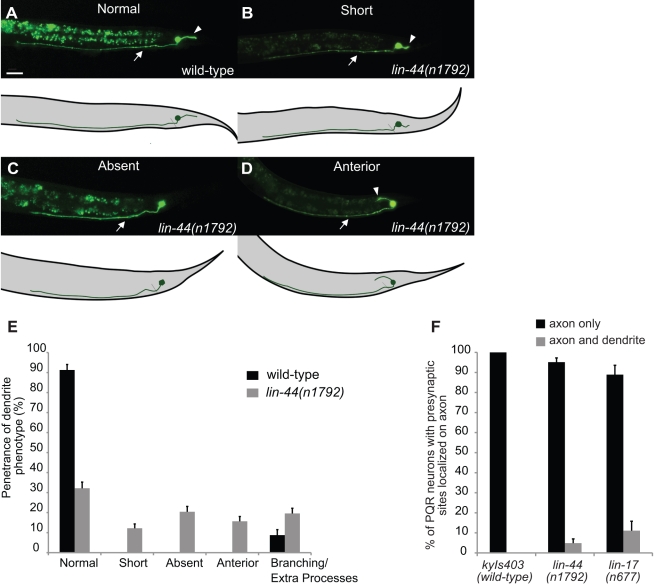
PQR dendrite defects in *lin-44/wnt* mutants. (A) Normal PQR morphology in a wild-type adult. Arrowhead indicates the dendrite and arrow indicates the axon in this and in the following panels. (B–D) *lin-44(n1792)* mutant animals with PQR dendrites that are short (B), absent (C), or anteriorly misrouted (D). Dendrite/axon branching and extra processes extending from the cell body were also observed in both wild-type and *lin-44(n1792)* animals (not shown). (E) Quantification of dendrite defects in *lin-44(n1792)* mutant animals. (F) Bar graph showing location of PQR presynaptic sites (visualized using *Pgcy-36::YFP::RAB-3*) in wild-type, *lin-44(n1792)*, and *lin-17(n677)* animals. *n* represents at least 100 animals for each data set. Error bars represent the s.e. of proportion. Scale bar: 20 µm.

PQR is an oxygen-sensory neuron with its cell body positioned in the posterior lumbar ganglion on the left side of the animal [26]. PQR extends a single axon anteriorly along the ventral nerve cord and a single dendrite posteriorly towards the tail (Figures 1D, 2A). The tip of the dendrite, which is part of the left phasmid sensory organ, protrudes with its sensory cilia into the pseudocoelom. PQR is born post-embryonically, facilitating investigation of its development in newly hatched larvae. A *gcy-36::GFP* reporter was used as a selective marker for PQR, allowing visualization of its dendrite during development, starting at the L1 stage (see Materials and Methods).

PQR arises as a descendant of the QL neuroblast, and subsequently migrates towards the tail. We observed that upon reaching its final destination, at 5.5–6.5 h after hatching, PQR assumed a rounded or elliptical shape, without any neurites (Figure 1A). At 6.5–7 h, dendrite formation began with lamellipodia-like extensions emerging on the dorsal-posterior region of the cell body, which had become elliptical or triangular in shape (Figure 1B). At this stage, no other projections were present, indicating that dendrite outgrowth is initiated before outgrowth of the axon. At 7–7.5 h, the dorsal-posterior protrusion thinned and extended into a developing dendrite with a growth cone at its distal tip, and the cell body became rounder in shape (Figure 1C). At the same time, the axon began to emerge from the ventral-anterior side of the cell, appearing as a small neurite that, unlike the dendrite, did not present a large growth cone at its tip. By 7.5 h, both the dendrite and axon were visible and continued to extend to their final positions until 18 h after hatching (L2/L3) (Figure 1D). PQR subsequently maintained its morphology throughout adulthood (Figure 2A). Overall, our analysis demonstrates that the PQR dendrite forms by growth cone crawling and is initiated prior to axon outgrowth.

### LIN-44/Wnt Regulates Dendrite Formation in PQR

We next used a candidate gene approach to discover the molecules regulating dendrite development in PQR. We found that animals mutant for LIN-44/Wnt presented severe defects, with PQR dendrites that were short, absent, or misrouted in the anterior direction (Figure 2B–D, and quantified in 2E). The axon, however, appeared morphologically normal. These defects could arise from a dendrite-specific effect or a change in neuronal polarity whereby the identity of the neurites is compromised. To distinguish between these two possibilities we investigated whether there were any changes in the location of the presynaptic sites of PQR, which are normally on the axon. *rab-3* encodes for a vesicle-associated Ras GTPase, which localizes to presynaptic densities [27],[28]. Using a YFP::RAB-3 fusion protein expressed specifically in PQR (*Pgcy-36::YFP::RAB-3*), we found that the presynaptic sites in *lin-44* mutants were largely located on the axon as in wild-type animals (Figure 2F). This suggests that the identity of the neurites is unchanged and that the PQR defect of the *lin-44* mutant is dendrite-specific.

Next, we tested if the PQR dendrite defect of *lin-44* mutant animals could arise from an abnormal cell division in the precursor cell. However, we found that the asymmetric cell divisions of the PQR precursor occurred normally in the *lin-44* mutant animals (Figure S1), precluding this possibility.

Finally, we investigated whether the absent and short dendrite phenotypes we observed were generated either by excessive pruning or by direct outgrowth failure. Examination of early stages of PQR development in *lin-44* mutants revealed that the dendrite often failed to form or fully extend (Table S1); we also observed animals with dendritic growth cones developing abnormally on the anterior side of the neuron, which would explain the anteriorly misrouted dendrites observed in adults (Table S1). Thus, our results indicate that LIN-44 acts at very early stages of PQR development by regulating proper formation of the growth cone and its extension.

### LIN-44/Wnt Acts as an Attractant Cue for the Developing PQR Dendrite

**Figure 3:**
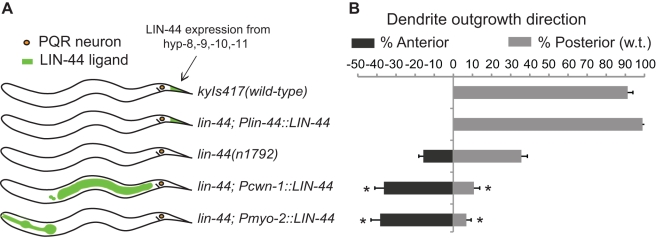
LIN-44 functions as an attractive cue. (A) Schematic diagram of normal LIN-44 expression posterior to PQR and ectopic expression of LIN-44 from different regions of the animal's body (indicated as shaded green areas). (B) Quantification of anteriorly directed dendrites and normal dendrites for animals with the corresponding LIN-44 expression patterns shown in (A). *n* represents at least 100 animals for each data set. Error bars represent the s.e. of proportion. Asterisks indicate difference from *lin-44(n1792)* animals, by Bonferroni *t* test, *p*<0.05.

The Wnt ligand LIN-44 is expressed in close proximity to the PQR neuron from four hypodermal cells (hyp-8, -9, -10, and -11) in the tip of the tail [29], a position posterior to the PQR dendrite (Figure 3A). As the PQR dendrite grows towards the source of LIN-44, we hypothesized that this molecule might act instructively as an attractive cue for the developing dendrite. Alternatively, LIN-44 may act as a permissive cue, whereby its positional information is not essential for correct dendrite development. To distinguish between these two possibilities, we expressed LIN-44 ectopically from regions anterior to the PQR cell body in *lin-44* mutant animals, using a version of LIN-44 genomic DNA that had been engineered to contain a secretion signal sequence to ensure proper secretion from cells that do not normally produce LIN-44 [16]. Transgenic lines were generated to express LIN-44 from the *myo-2* promoter [30] in the pharynx (*Pmyo-2::LIN-44*), or from a short fragment of the *cwn-1* promoter [21] in the intestine and head neurons (*Pcwn-1::LIN-44*) (Figure 3A and Figures S2, S3). When compared to *lin-44* mutant animals, transgenic animals expressing LIN-44 anterior to PQR displayed a decrease in the proportion of normal dendrites and an increase in the proportion of dendrites that were misrouted in the anterior direction, towards the ectopic source of LIN-44 (Figure 3B and Figures S2, S3). On the contrary, expression of LIN-44 from its endogenous promoter (*Plin-44::LIN-44*) provided strong rescue of the PQR dendrite defect of *lin-44* mutant animals (Figure 3B).

We next examined the ectopic expression of LIN-44 from the *myo-2* promoter in the wild-type background and found that it altered the normal development of the PQR dendrite (Figure S4). Thus, the worsening of dendrite defects observed when LIN-44 is ectopically expressed from anterior regions suggests that LIN-44 has an instructive role in PQR dendrite development, whereby it acts as an attractive cue to direct the outgrowth of the dendrite.

### A Pattern of LIN-44 Necessary for Dendrite Outgrowth Is Established During Embryogenesis

**Figure 4:**
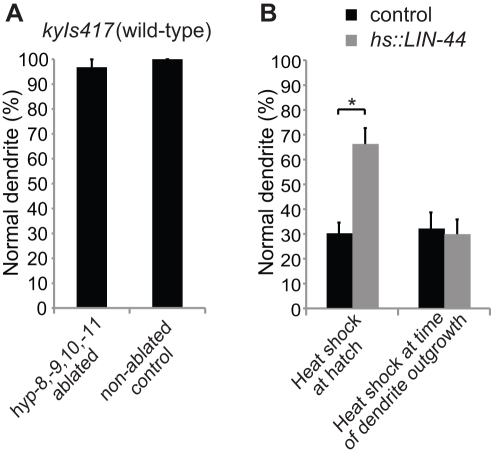
LIN-44 expression is necessary and sufficient prior to dendrite development. (A) Percentage of normal dendrites in wild-type animals with hypodermal cells hyp-8, -9, -10, and -11 ablated at hatching, compared to non-ablated controls. (B) Rescue of dendrite defects in *lin-44(n1792)* by heat shock induced LIN-44 expression in larvae at hatching but not at the time of dendrite outgrowth. Dendrite defects were scored at the L4 stage and were compared to non-transgenic control siblings (see Materials and Methods). Error bars represent the s.e. of proportion. For each data set, *n* represents at least 30 animals in (A), and 47 animals in (B). Asterisk indicates values different from controls by Student's *t* test, *p*<0.05.

In wild-type *C. elegans*, the four tail hypodermal cells hyp-8, -9, -10, and -11 express LIN-44 throughout embryogenesis and larval stages [29]. In order to define the time period in which LIN-44 is required for normal PQR dendrite development we eliminated larval production of LIN-44 by laser ablation of the hyp-8, -9, -10, and -11 hypodermal cells. Remarkably, in adult animals that were laser-ablated as newly hatched L1 larvae, the PQR dendrite appeared to be largely unaffected (Figure 4A) even though the ablations were performed several hours before PQR is born in the mid-L1 stage. This result indicates that LIN-44 expression from these hypodermal cells during embryogenesis is sufficient for the correct development of the PQR dendrite.

To further define the temporal requirement of LIN-44 we next utilized an inducible heat shock promoter to express LIN-44 (*Phsp16-2::LIN-44*) in a *lin-44* mutant background at specific times during development. Heat shock-induced LIN-44 expression in newly hatched L1 animals partially rescued PQR dendrite defects (Figure 4B and Figure S5). However, when animals were heat shocked later, at the time of dendrite outgrowth, no such rescue effect was observed (Figure 4B), suggesting that LIN-44 expression is required prior to PQR dendrite outgrowth.

The *hsp16-2* promoter drives expression broadly throughout the body of the animal, in cells that are both anterior and posterior to PQR [31]. Thus, the dendrite rescue we observed in heat shocked animals could indicate that LIN-44 plays a permissive role, or that the ligand is produced more efficiently from regions posterior to PQR. To further investigate this we expressed *Phsp16-2::LIN-44* into a wild-type background and found that the ectopic expression of LIN-44 generated PQR defects similar to those of *lin-44* mutants, confirming the instructive role of this molecule (Figure S6). Taken together, these results suggest that a molecular pattern of LIN-44 generated prior to PQR formation, during embryonic development and early L1, is both necessary and sufficient to instruct PQR dendrite outgrowth hours later, at which time the source of LIN-44 expression becomes dispensable.

### LIN-17/Frizzled May Act as a Receptor for LIN-44

**Figure 5:**
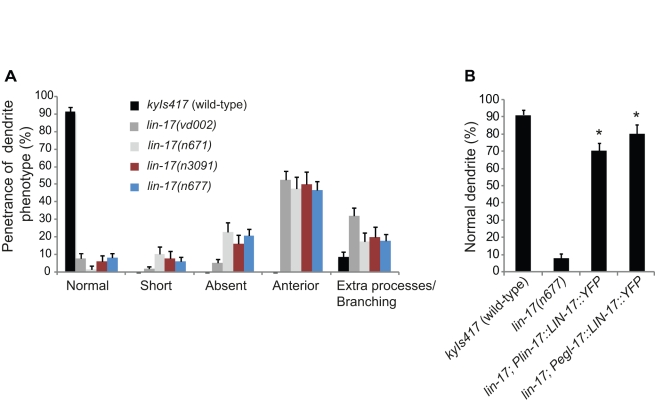
*lin-17* regulates dendrite development cell-autonomously in PQR. (A) Quantification of dendrite defects in different *lin-17* mutant alleles. (B) Transgenic rescue of PQR dendrite defects in *lin-17(n677)* mutants by expression of LIN-17 cDNA tagged with YFP driven by the *lin-17* promoter (*Plin-17::LIN-17::YFP*) or the *egl-17* promoter (*Pegl-17::LIN-17::YFP*). In both panels (A and B), only animals with correctly positioned PQRs were scored. Error bars represent the s.e. of proportion. *n* represents at least 50 animals for each data set. Asterisks indicate values different from *lin-17(n677)* mutants, Bonferroni *t* test, *p*<0.05.

LIN-17 is a Frizzled molecule known to function as a receptor for LIN-44 in a variety of developmental processes [14],[16],[17],[29],[32]–[35]. We found that *lin-17* mutants had defects resembling those of *lin-44*, with PQR dendrites that were short, absent, and misrouted anteriorly (Figure 5A). *lin-17* mutants also presented a strong migration defect [18],[19], with a high percentage (60% to 90%) of PQR neurons mispositioned in anterior regions of the body. Thus, our analysis was performed on those animals in which PQR was correctly positioned in order to eliminate any possible effect that the aberrant location may have had on PQR dendrite development. Importantly, *lin-17* mutants, like *lin-44* mutants, appeared to have largely normal localization of presynapses to the axon, as visualized using the YFP::RAB-3 fusion protein expressed specifically in PQR (*Pgcy-36::YFP::RAB-3*), eliminating the possibility of a switch in neurite identity (Figure 2F).

In addition to testing known alleles of *lin-17*, we also performed a forward genetic screen and isolated a previously uncharacterized allele, *vd002*, consisting of a G to A transition in position 490 of the *lin-17* gene that resulted in a cysteine residue being replaced by a tyrosine residue (Figure 5A). The isolation of this mutant from an unbiased screen further supports the significance of *lin-17* in this process.

**Figure 6:**
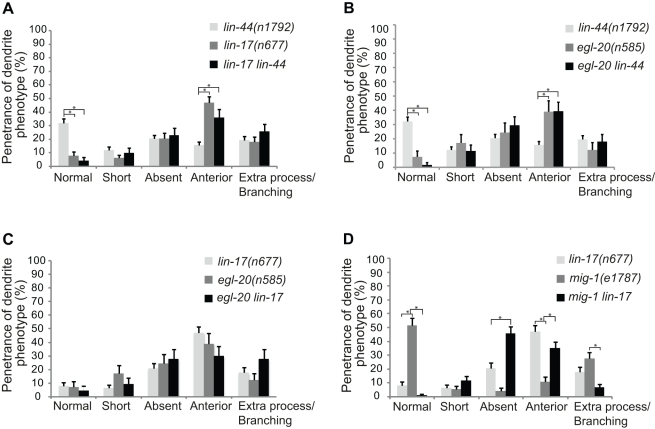
Dendrite defects in Wnt and Frizzled mutants. Quantification of dendrite defects in single and in double mutants; *lin-17 lin-44* (A); *egl-20 lin-44* (B); *egl-20 lin-17* (C); and *mig-1 lin-17* (D). All data shown were obtained from animals in which PQR was correctly positioned. *n* represents at least 40 animals for each data set. Error bars represent the s.e. of proportion.

To investigate whether there might be a genetic interaction between *lin-17* and *lin-44* with respect to PQR dendrite development, we next examined *lin-17 lin-44* double mutants and found that the dendrite defects were qualitatively and quantitatively similar to those of *lin-17* mutants (Figure 6A). This indicates that these two molecules function in the same genetic pathway with respect to PQR dendrite development and strongly suggests that LIN-44 acts as a ligand for LIN-17 in this process.

### LIN-17 Acts Cell-Autonomously in the PQR Neuron Prior to Dendrite Outgrowth

LIN-17 is expressed extensively and dynamically in several cells of the tail region including PQR (Figure S7) [35]. Wnt signaling through the LIN-17 receptor could occur cell-autonomously within PQR or could result from interactions with the surrounding cells. We first tested whether LIN-17 acts cell-autonomously by expressing the wild-type *lin-17* cDNA from the *gcy-36* promoter, which is transcriptionally active in PQR during the final stages of its migration. This transgene failed to rescue the dendrite defects, despite being tested at a range of different concentrations (see Materials and Methods). We therefore questioned whether LIN-17 might be required in PQR at earlier stages, before the *gcy-36* promoter is transcriptionally active. To test this possibility we used the *egl-17* promoter that is highly and selectively expressed in the precursors of PQR during the L1 stage [36],[37] to drive LIN-17 expression from the time PQR was born. Wild-type LIN-17 cDNA expressed by the *egl-17* promoter (*Pegl-17::LIN-17::YFP*) strongly rescued the PQR dendrite defects of *lin-17* mutants, to levels similar to that of the endogenous promoter (*Plin-17::LIN-17::YFP*) (Figure 5B). These results suggest that LIN-17 regulates dendrite development in a cell-autonomous fashion and is required very early in development, before or during PQR migration.

The PQR dendrite is ensheathed by PHso2L, a glia cell of the left phasmid sensillum; this sensillum comprises two socket cells (PHso1L, PHso2L), a sheath cell (PHshL), and two sensory neurons (PHAL and PHBL) [26]. Recent results in different systems have demonstrated a role of the support cells in regulating dendrite development [4],[7]. To determine if similar mechanisms were in place for PQR development, we next performed cell-ablation experiments whereby we selectively eliminated the socket cells or the socket cells together with the sheath cells. PQR morphology in ablated animals was largely normal, with only a small number of animals presenting short dendrites when left and right phasmid socket cells were ablated (3/15) or when left phasmid socket and left sheath cells were ablated (2/19). We never observed the penetrance and variety of defects of the *lin-17* mutants. These results indicate that glial cells play a minor role in only the final stages of dendrite extension and suggest that LIN-17 does not have an effect on the PQR dendrite through these support cells (Table S2). In addition, ablations of the phasmid neurons PHA and PHB also had no effect on PQR dendrite development (Table S2), thereby providing further evidence that the function of LIN-17 in PQR dendrite development is unlikely to be mediated by the surrounding cells.

To further understand how LIN-17 acts on the PQR dendrite, we then asked at what stage in PQR development LIN-17 was visible on the cell membrane and how LIN-17 was distributed in PQR. Using a LIN-17::YFP functional fusion protein expressed under the control of the *egl-17* promoter, we observed faint, relatively uniform localization of LIN-17 on the membrane of the QL.a cell as it was dividing into QL.aa and PQR (unpublished data). Following this division, the membrane-localized LIN-17::YFP in PQR decreased until it was barely visible at the time at which PQR had completed its posterior migration (unpublished data). This reduction in LIN-17::YFP appeared to be independent of down-regulation by the *egl-17* promoter and is consistent with our previous results suggesting an early role for LIN-17 in regulating PQR dendrite outgrowth. We suggest that ubiquitous membrane-localization of LIN-17 may be required to detect the posterior source of Wnt ligand, which acts as the directional signal for the PQR dendrite.

### Multiple Wnt and Frizzled Genes Regulate Dendrite Development

Multiple Wnt ligands and Frizzled receptors are known to function in basic developmental processes in *C. elegans* and have frequently been shown to have redundant or synergistic roles. Although *lin-44* mutants present striking PQR dendrite defects, 32% of these animals still have the ability to sprout a normal PQR dendrite, suggesting the involvement of other molecules in this process. We therefore tested three other Wnt molecules–EGL-20, CWN-1, and CWN-2–for possible roles in PQR dendrite formation. EGL-20 is expressed around the PQR cell body, in a group of epidermal and muscle cells near the anus [13],[20], and CWN-1 and CWN-2 are expressed to a greater extent anteriorly in the intestine, body wall muscle, and neurons in the midbody and head regions, anterior to the PQR cell body [13],[22],[38]. No significant dendrite defects were observed in *cwn-1* or *cwn-2* single mutants. The *cwn-1 cwn-2* double mutant presented a higher percentage of ectopic processes from the cell body, and dendrite branching, compared to the single mutants, but no absent-dendrite or dendrite-misrouting defects were observed (Table S3). This suggests that these molecules are less directly involved in development of the PQR dendrite, but are important to prevent the formation of ectopic processes. Although the loss of *cwn-1* alone caused no significant dendrite defects on PQR, when combined with the *lin-44* mutation it was able to enhance the dendrite misrouting defects of *lin-44* mutants (Table S3). Thus, CWN-1 might have a minor and redundant role in PQR dendrite development.

As previously described, *egl-20* mutants have a very strong Q cell migration defect [18]–[20] resulting in 97%–98% of animals having anteriorly positioned PQR neurons. Restricting our analysis to those animals with PQR correctly positioned, we found that only 7% of *egl-20* animals developed a normal, full-length dendrite, whereas the rest presented qualitatively similar defects to those of *lin-44* and *lin-17* animals, with absent, short, and anteriorly misrouted PQR dendrites (Figure 6B). *egl-20* mutants presented a higher proportion of anterior dendrites, as compared to *lin-44* mutant animals (Figure 6B), but the PQR dendrite phenotype of the *egl-20 lin-44* double mutant did not display a significant worsening of defects when compared to the *egl-20* single mutant. This suggests that *egl-20* and *lin-44* may interact to regulate PQR dendrite formation (Figure 6B). Furthermore, the *egl-20 lin-17* double mutant was no worse than either of the single mutants (Figure 6C), suggesting that LIN-17 may act as a receptor for both EGL-20 and LIN-44.

Taken together, the above results indicate that *egl-20* and *lin-44* are the major regulators of PQR dendrite outgrowth, and appear to genetically interact, whereas *cwn-1* plays only a minor role in the process. To determine the possible roles of other Frizzled receptors, we also studied PQR dendrite formation in *cfz-2* and *mig-1* mutants. *cfz-2* mutants showed no significant defects, whereas *mig-1* mutants presented 50% normal PQR dendrite (Figure 6D, Table S3). Thus, LIN-17 appears to be the main Frizzled receptor regulating PQR dendrite formation. To analyze functional redundancy among the Frizzleds, we tested whether *mig-1* could enhance the *lin-17* defect. In the *mig-1 lin-17* double mutant, there was almost a 2-fold increase in the absent-dendrite phenotype (Figure 6D), indicating a possible parallel role of *mig-1* in PQR dendrite formation.

## Discussion

Dendrites, the specialized structures that allow neurons to receive sensory information from the environment and to relay signals to one another, must develop properly in order to build a functioning nervous system. Recent reports of dendrite morphogenesis have advanced our understanding of dendrite sculpting and arborization [1],[6],[11],[12],[39], neuronal polarity [3],[11],[40], dendrite extension [4],[5], and dendrite orientation [2],[7]. To our knowledge, our study is the first to demonstrate that the initial outgrowth of a dendrite in vivo is controlled by Wnts and Frizzleds. Mutations in the Wnt ligands LIN-44 and EGL-20 and in the Frizzled receptors LIN-17 and MIG-1 cause a failure in dendrite development, resulting in dendrites that are absent, short, or misrouted. Our findings demonstrate that the Wnt ligand LIN-44 instructs the development of the dendrite through an attractive mechanism and is required prior to the initiation of dendrite outgrowth. This effect is likely to be mediated through the LIN-17 receptor, which acts cell-autonomously in PQR.

### A Spatial Pattern of LIN-44/Wnt Established During Embryogenesis Acts as an Attractant for the Developing PQR Dendrite

Several studies across different model systems have shown that Wnts can act instructively as both attractants and repellents in neurodevelopmental processes such as axon guidance, synapse formation, and neurite outgrowth [13],[16],[17],[23],[41]–[43]. Conversely, Wnt molecules can also act in a permissive manner, as non-spatial cues [14],[15],[20],[22].

Our results suggest that posteriorly expressed LIN-44 acts as an attractive cue for the PQR dendrite. Ectopic expression of LIN-44 from the anterior side of PQR increases the tendency for dendrites to emerge and grow anteriorly, towards the source of LIN-44. This role of LIN-44 as an attractant in PQR dendrite development differs from its role as a repellent signal for synaptic clustering in the dorsal section of the DA9 motor neuron [16], highlighting the distinct effect of LIN-44 on these neighbouring neurons.

The partial rescue of PQR dendrite defects by ubiquitous expression of LIN-44 from the heat shock promoter could suggest a permissive role for LIN-44. However, a possible alternative interpretation is that local asymmetry of the ligand is generated, providing rescue when the concentration is higher on the posterior side of PQR. This conclusion is supported by the observations that a higher concentration of ligand (increased length of heat shock) is unable to increase the rescue, and that in the wild-type background heat shock-directed expression causes dendrite defects. To be fully functional, Wnts must undergo post-translational modifications, sorting in the endoplasmic reticulum, and secretion from the cells where they are expressed [44]. It is possible that cells that do not normally express LIN-44 have lower efficiency in regulating the proper maturation and secretion of this Wnt molecule. Hence LIN-44 expression from the heat shock promoter may provide functional, secreted LIN-44 with variable efficiency depending on the tissue of expression.

Wnt patterning occurs during embryogenesis, at a time when many neurons are born. Our observation that PQR forms a normal dendrite following ablation of the tail hypodermal cells at the time of hatching suggests that embryonically expressed LIN-44 provides spatial information needed by the developing PQR several hours later. However, PQR remains receptive to heat shock misexpression of LIN-44 up until the dendrite begins developing. It is not known how stable Wnts are in *C. elegans*; however, in *Drosophila* the Wnt Wingless (Wg) and the morphogen Decapentaplegic (Dpp) are stable for about 3 h [45],[46]. Wnts can also function at long distances. In *C. elegans*, for example, EGL-20 has been shown to direct cell migration across half the animal's body length [20],[46]. Similarly in *Drosophila*, Wg can cover 10–20 cell diameters away from its source in the developing wing [47],[48] spreading over a distance of about 50 µm in 30 min [46]. Our results showing an effect of LIN-44 when expressed in the pharynx from the promoter *myo-2* in a region far from PQR also suggest a potential long range effect for this ligand.

### LIN-17/Frizzled Regulates PQR Dendrite Development in a Cell-Autonomous Manner

Emerging evidence suggests that dendrites of sensory neurons are shaped in a variety of ways. In contrast to dendrite development by retrograde extension, or towing by associated cells [4],[7], we and others [49] have observed that the dendrite of PQR forms by growth cone crawling, a mode of development more commonly seen in axons. In LIN-44 mutants, this growth cone often fails to form, preventing the outgrowth of a dendrite. Our results demonstrate that LIN-17, a receptor for LIN-44, cell-autonomously regulates the initiation and outgrowth of the PQR dendrite. To our knowledge, a ligand-receptor pair that can specifically affect the development of a dendrite in this manner has not previously been described.

Interestingly, phasmid glia associated with the PQR dendrite do not have a major effect on its development. It has previously been shown that *lin-44* and *lin-17* mutants have defects in phasmid socket glia that arise due to disrupted polarity of the T cell precursor [29],[35],[50]. However, the aberrant structure of the phasmid in these mutants does not seem to be the main cause of dendrite defects, as ablation of these cells did not reproduce the mutant phenotypes. Notably, glia appear to have some involvement in the final extension of the dendrite, as some ablated animals had short dendrites. This is reminiscent of a previous study in which it was demonstrated that ablation of the sheath glia associated with the CEP sensory neuron in the head of C. *elegans* resulted in a failure of the sensory dendrite of this neuron to fully extend [51].

**Figure 7:**
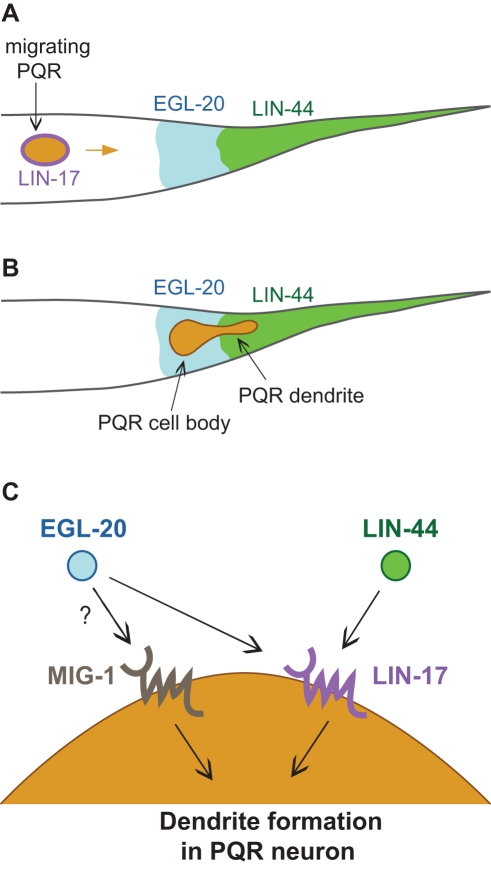
Cellular and genetic models for PQR dendrite development. (A) As PQR migrates towards the tail, the LIN-17 receptor on the cell surface detects posteriorly expressed EGL-20 and LIN-44; the distance between the PQR neuroblast and the LIN-44 source is approximately 35 µm. (B) Once migration is completed, PQR extends its dendrite towards the attractive source of LIN-44. (C) The Wnt ligands LIN-44 and EGL-20 both act through the LIN-17/Frizzled receptor expressed in PQR to regulate dendrite formation. MIG-1/Frizzled also contributes to proper dendrite formation through a parallel pathway.

Different lines of evidence suggest that LIN-17, like LIN-44, may be required early in development to promote normal dendrite outgrowth. Cell-specific LIN-17 expression can rescue *lin-17* dendrite defects if induced very early, before PQR is born, but has no such effect when induced later, once the cell has almost completed its migration. Furthermore, LIN-17::YFP expression from the rescuing *egl-17* promoter appeared to become extremely faint or absent by the time the dendrite began to develop. This raises the interesting possibility that levels of LIN-17 receptor on the PQR cell surface are temporally regulated to elicit the appropriate response to Wnt ligands. We propose a model in which the LIN-17 receptor, present at low levels on the membrane of the PQR cell from the moment it is born, detects a posterior source of LIN-44 that signals the dendrite to emerge from the posterior side of the cell (Figure 7A,B). This initial specification of the site of dendrite outgrowth appears to be an important determinant of the subsequent direction of dendrite outgrowth. The tendency for *lin-44* and *lin-17* mutant dendrites to grow anteriorly from the PQR cell, rather than from random orientations (including dorsal or ventral), may imply the presence of an intrinsic anterior-posterior bias of the site and direction of PQR dendrite outgrowth controlled by Wnts and Frizzleds, or the existence of a dorso-ventral dendrite outgrowth controlled by other guidance molecules still unknown.

### Multiple Wnt and Frizzled Molecules Coordinate Development of the PQR Dendrite

In *C. elegans*, Wnts are expressed in different regions along the anterior-posterior axis. These different Wnts have often been shown to have distinct effects on cells that are located in proximity to the respective source of Wnt expression. Our genetic studies suggest that, similar to LIN-44, the posteriorly expressed Wnt ligand EGL-20 also acts through the LIN-17 receptor to regulate PQR dendrite development (Figure 7C), which could explain why *lin-17* defects are more severe than those of *lin-44*. However, whether EGL-20 plays an instructive role in this process remains unclear. Previous studies have also shown that both LIN-44/Wnt and EGL-20/Wnt can function through LIN-17/Frizzled; however, whether Frizzled receptors can simultaneously bind multiple Wnts, or whether Wnts can form homo- or hetero-dimers, remains unknown.

The Wnt molecules CWN-1 and CWN-2 are both expressed more broadly in the body wall muscle, intestine, ventral cord neurons, and some head neurons [13],[21],[22],[38]. Although these Wnts do not appear to directly regulate PQR dendrite development, our observation that a significant proportion of *cwn-1* and *cwn-2* mutants present ectopic processes on PQR suggests an indirect role in neurite pruning. This is consistent with recent findings that identify CWN-1 and CWN-2 as key regulators of developmental pruning of the head neuron AIM [21].

The MIG-1 receptor appears to act synergistically in a parallel pathway to LIN-17 (Figure 7C). Notably, the increase in the percentage of the absent dendrite phenotype of the *lin-17 mig-1* double mutant compared with the *lin-17* mutant suggests a role for MIG-1 in regulating the ability of the neuron to send out a dendrite, regardless of its direction.

Wnt morphogens have diverse functions in developmental processes across species, yet how they act with such precision on a single cell within a closely wired nervous system remains enigmatic. As we and others have shown, spatio-temporal organization of Wnts and their Frizzled receptors must be tightly orchestrated. The challenge now will be to gain insight into how these molecules are patterned and how they can be interpreted differently by individual cells.

## Materials and Methods

### Strains and Genetics

Nematodes were cultured using standard methods [52]. All experiments were performed at 18°C except where otherwise noted. The following mutations were used: LGI, *lin-17(n677)*, *lin-17(n671)*, *lin-17(n3091)*, *lin-17(vd002)*, *lin-44(n1792)*, *mig-1(e1787)*; LGII, *cwn-1(ok546)*; LGIV, *egl-20(n585)*, *cwn-2(ok895)*; LGIV, *cfz-2(ok1201)*. Transgenes used were: *kyIs417[Pgcy-36::GFP, Podr-1::dsRed]*, *kyIs403[Podr-1::dsRed2, Pflp-18::UNC-43g::dsRed2, Pgcy-36::YFP::RAB-3, Pgcy-36::mCFP]*, *vdEx127[Phsp16-2:LIN-44 (10 ng/µl), Pcoelomocyte::GFP (25 ng/µl)]*, *wyEx806[Plin-44::signal sequence:: flag::GFP::lin-44 genomic coding::lin-44 3′UTR, odr-1::GFP]*, *vdEx224[Pcwn-1::signal sequence::flag::GFP::lin-44 genomic coding (20 ng/µl), Pcoelomocyte::GFP (30 ng/µl)]*, *vdEx235(Pmyo-2::signal sequence::flag::GFP::lin-44 genomic coding (20 ng/µl), Pcoelomocyte::GFP (30 ng/µl)]*, *vdEx251[Podr-1::dsRed (30 ng/µl), Pegl-17::LIN-17::YFP (20 ng/µl), Pgcy-36::mCherry (0.5 ng/µl)]*, *vdEx133[Plin-17::LIN-17::YFP (10 ng/µl), Pchs-2::dsRed (2 ng/µl),* pSM *(10 ng/µl)]*, *vdEx265[ Plin-17::mCherry (20 ng/µl), Pegl-17::GFP (50 ng/µl)].* The *kyIs417* strain was generated in Cori Bargmann's lab, the *kyIs403* strain was provided by Manuel Zimmer and Cori Bargmann, and the *wyEx806* strain was provided by Kang Shen.

### Molecular Biology

Standard molecular biology methods were used. All constructs were cloned into pSM (a kind gift from Steve McCarroll and Cori Bargmann), a derivative of pPD49.26 (Andrew Fire). The *Pmyo-2::GFP::LIN-44* and *Pcwn-1::GFP::LIN-44* constructs were generated by cloning a *myo-2* promoter and a 170 bp fragment of the *cwn-1* promoter [21] into FseI/AscI sites of pSM. A sequence encoding *signal sequence::flag::GFP::LIN-44 genomic DNA* (modified from the *wyEx806* transgene [16]) was cloned downstream of each promoter into BamHI/NheI sites.

The *Plin-17::LIN-17::YFP* rescue plasmid was generated by inserting a HindIII/NheI digested 6.5 kb fragment of the *lin-17* promoter upstream of a *LIN-17::YFP* clone (*Pitr-1 pB::LIN-17::YFP* [a gift from Kang Shen]).

The *Pegl-17::LIN-17::YFP* rescue plasmid was made by inserting a 5.4 kb NotI/FseI digested fragment of the *egl-17* promoter upstream of *LIN-17::YFP* (digested from *Plin-17::LIN-17::YFP* plasmid and cloned into NheI/PspOM1 sites).

*Pegl-17::mCherry* was generated by digesting mCherry from a pSM *mCherry* clone and inserting into KpnI/PspOMI sites behind the *egl-17* promoter. *Pgcy-36::mCherry* was created by inserting 1.1 kb *gcy-36* promoter into pSM *mCherry*. The *Plin-17::mCherry* clone was generated by cloning a BamHI/NheI digested 6.5 kb fragment of the *lin-17* promoter into pSM *mCherry*.

A *Pgcy-36::LIN-17::YFP* expression construct was unable to rescue dendrite defects in *lin-17(n677)* mutants when injected at concentrations of 0.2, 0.5, 1, 2, and 10 ng/µl.

### Analysis of PQR Morphology

We analyzed PQR development in synchronized populations of anesthetized larvae (L1 stage) in a *kyIs417*(*Pgcy-36::GFP*) background. Animals were synchronized by collecting newly hatched animals, from a plate containing only eggs, every 10 min using M9 buffer. Synchronized animals were transferred to fresh plates and grown for 5–9 h at 22°C before imaging. Developmental stages were characterized in synchronized populations, with little variation among animals.

PQR morphology was scored at L4 or adult stages. Mutations in *mig-1*, *lin-17*, and *egl-20* caused PQR migration defects, resulting in anterior (and in some cases posterior) mis-positioning of PQR. Given that this would cause PQR to be in a different position in relation to its normal surroundings, and importantly the source of LIN-44, we chose to score dendrite defects only in those animals where PQR had migrated to its normal position. The PQR dendrite was scored as short if it was less than three cell bodies in length.

### Heat Shock Experiments

Wild-type and *lin-44(n1792)* mutant animals carrying the *Phsp16-2::LIN-44* transgene were maintained at 18°C. As development at this temperature is slower than at 22°C as in Figure 1, dendrite outgrowth occurs at ∼8 h rather than ∼6.5 h. L1 animals were heat shock-induced at 33°C in a water bath for 30 min (or longer, where indicated) at different stages of development as indicated, following which they continued to grow at 18°C. Transgenic animals (*lin-44; Phsp16-2::LIN-44*) and non-transgenic controls (*lin-44*) were scored at the L4 stage or as young adults.

### Microscopy and Cell Ablations

Animals were mounted on 4% agar pads and immobilized using tetramisole hydrochloride (0.01%–0.03%). Epifluorescence was used to visualize animals with a Zeiss Axioimager Z1 and a Zeiss Axioimager A1 microscope. A Photometrics camera, Cool snap HQ^2^, was used for imaging. Metamorph software was used to analyze the collected Z stacks. Developing stages of PQR were imaged using a Zeiss LSM510META confocal microscope and Zen 2008 software. An antifading agent, Dayco, was used in addition to tetramisole hydrochloride.

Laser ablations were performed in L1 animals carrying the *kyIs417* transgene using a MicroPoint Laser System Basic Unit attached to a Zeiss Axio Imager A1 (Objective EC Plan-Neofluar 100×/1.30 Oil M27). Animals were ablated 0–1 h after hatching and were scored at the L4 stage. For ablations of phasmid glia and phasmid neurons, ablation success was determined at the L4 stage by soaking animals in DiI on slides for 2 h prior to scoring (DiI stains the phasmid neurons when these cells and the phasmid structure are unaltered [53]–[55]).

### Statistical Analyses

Statistical analyses were performed using Primer of Biostatistics 3.01. Error of proportions was used to estimate variation within a single population. The Student's *t* test was used in all cases, except in those with multiple comparisons, for which the Bonferroni *t* test was used.

## Supporting Information

Figure S1 **Cell division of the PQR precursors appear normal in** ***lin-44*** **mutant animals.** PQR precursors were visualized with the *egl-17::GFP* transgene. Two successive divisions of the Q cell descendants that give rise to PQR are shown for wild-type and *lin-44(n1792)* mutants: (A, B) division of the QL cell into QL.a and QL.p and (C, D), and a subsequent division that gives rise to QL.ap (PQR, white arrowhead) and an apoptotic sister cell (white arrow). (E, F) QL.ap (PQR) migrates posteriorly. Scale bar: 10 µm.
(EPS)

Figure S2 **Ectopic LIN-44 expression from anterior regions in** ***lin-44*** **mutants.** Quantification of normal and anteriorly directed dendrites in *lin-44* animals carrying the *Pcwn-1::LIN-44::GFP* transgene (A, B) or the *Pmyo-2::LIN-44::GFP* transgene (C, D). Independent transgenic strains (lines) are indicated with the concentration at which the transgene was injected. Error bars represent the s.e. of proportion. *n* is indicated underneath each data point. Asterisks indicate differences compared to non-transgenic controls, Student's *t* test, *p*<0.05.
(EPS)

Figure S3 **LIN-44 expressed from** ***lin-44, cwn-1*** **and** ***myo-2*** **promoters in** ***lin-44*** **mutant animals.** Images show LIN-44::GFP driven from the *lin-44* promoter in the tail hypodermal cells (A), the *cwn-1* promoter in the intestine (B) and the *myo-2* promoter in the pharynx (C). The *Plin-44::LIN-44::GFP* transgenic line was a kind gift from Kang Shen and has been previously visualized (Klassen and Shen, 2007). Scale bars: 10 µm (A and B) and 25 µm (C).
(EPS)

Figure S4 **LIN-44 ectopic expression from the** ***myo-2*** **promoter in wild-type animals.** Quantification of dendrite phenotypes in wild-type animals carrying the *Pmyo-2::LIN-44::GFP* transgene. Error bars represent the s.e. of proportion. Asterisk indicates difference compared to non-transgenic controls, Student's *t* test, *p*<0.05. *n* represents at least 100 animals for each data set.
(EPS)

Figure S5 **Rescue of** ***lin-44*** **dendrite defects with** ***hsp16-2*** **promoter driving LIN-44.** Quantification of dendrite phenotypes in *lin-44* animals with *hsp16-2::LIN-44* expression induced by a 30 min heat shock at hatching. Error bars represent the s.e. of proportion. *n* represents at least 50 animals for each data set.
(EPS)

Figure S6 **Ectopic LIN-44 expression from the** ***hsp16-2*** **promoter affects dendrite development in wild-type animals.** (A) Quantification of dendrite phenotypes in wild-type animals with *hsp16-2::LIN-44* expression induced by a 30 min heat shock at different stages of development. Animals were scored as adults and compared to non-transgenic controls (B). Development was analyzed at 18°C. At 6.5 h, PQR was completing migration. At approximately 7.5h, the first signs of dendrite outgrowth could be observed. By 8.5 h the dendrite had emerged. Error bars represent the s.e. of proportion. *n* represents at least 100 animals for each data set.
(EPS)

Figure S7 **LIN-17 is expressed in PQR.** PQR was visualized with *Pegl-17::GFP* (A, green), LIN-17 expression was visualized with *Plin-17::mCherry* (B, red). (C) Overlay of both images, visible in yellow, shows that LIN-17 is expressed in the PQR neuron. Scale bar: 10 µm.
(EPS)

Table S1 Dendrite defects in *lin-44* mutants at early stages of development.
(PPT)

Table S2 Dendrite phenotypes in cell-ablation experiments.
(PPT)

Table S3 PQR dendrite defects in single, double, and triple mutants.
(PPT)
