# Supplementary material for: LIN-44/Wnt Directs Dendrite Outgrowth through LIN-17/Frizzled in C. elegans Neurons
Source: PLoS Biol. 2011 Sep 20;9(9):e1001157. doi: 10.1371/journal.pbio.1001157 (PMC3176756; doi:10.1371/journal.pbio.1001157)
Supplement: Table S1 — Dendrite defects in lin-44 mutants at early stages of development. (PPT) [file pbio.1001157.s008.ppt]

## Slide 1
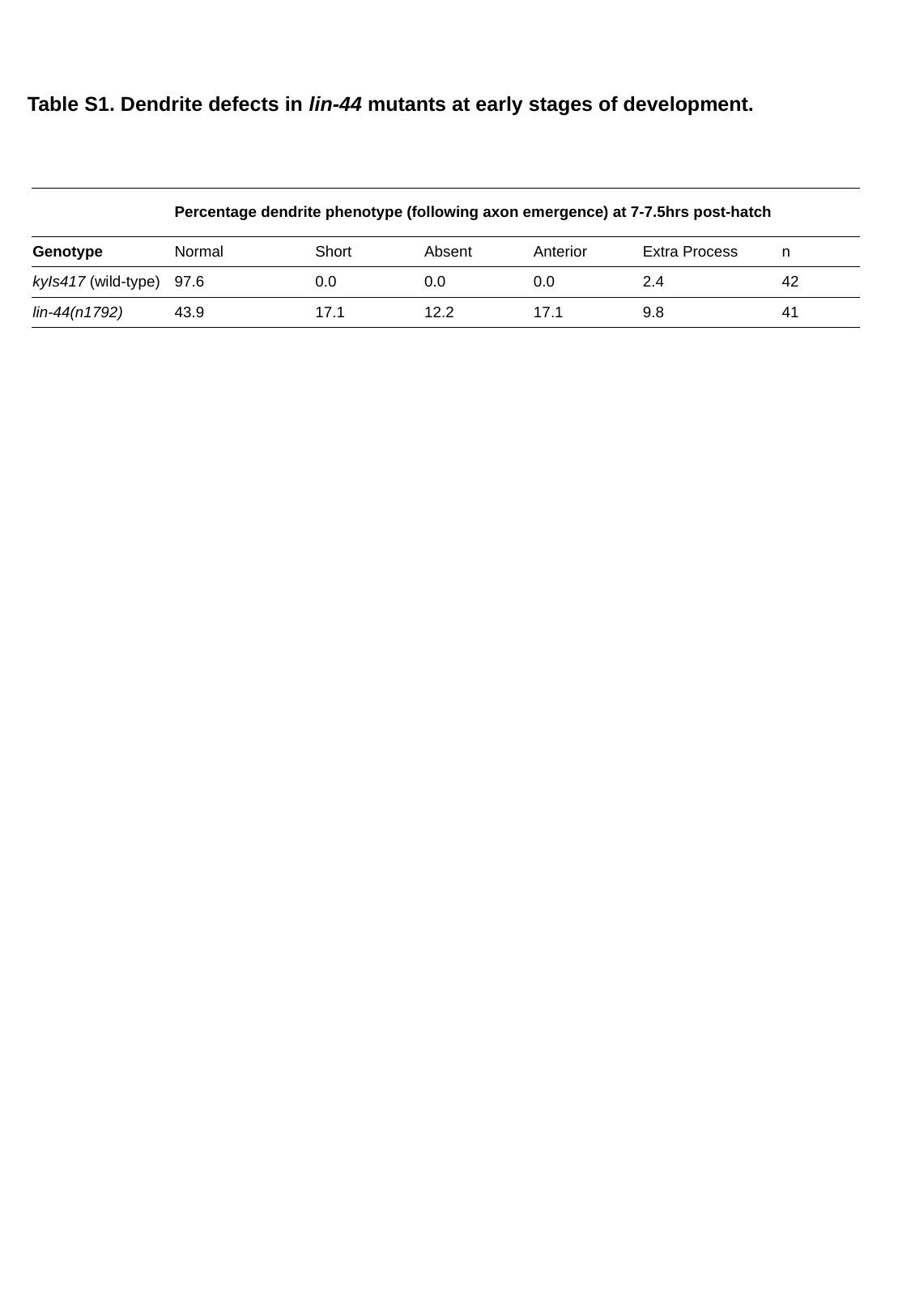

Table S1. Dendrite defects in lin-44 mutants at early stages of development.
| | Percentage dendrite phenotype (following axon emergence) at 7-7.5hrs post-hatch | | | | | |
| --- | --- | --- | --- | --- | --- | --- |
| Genotype | Normal | Short | Absent | Anterior | Extra Process | n |
| kyIs417 (wild-type) | 97.6 | 0.0 | 0.0 | 0.0 | 2.4 | 42 |
| lin-44(n1792) | 43.9 | 17.1 | 12.2 | 17.1 | 9.8 | 41 |
