# Supplementary material for: LIN-44/Wnt Directs Dendrite Outgrowth through LIN-17/Frizzled in C. elegans Neurons
Source: PLoS Biol. 2011 Sep 20;9(9):e1001157. doi: 10.1371/journal.pbio.1001157 (PMC3176756; doi:10.1371/journal.pbio.1001157)
Supplement: Table S2 — Dendrite phenotypes in cell-ablation experiments. (PPT) [file pbio.1001157.s009.ppt]

## Slide 1
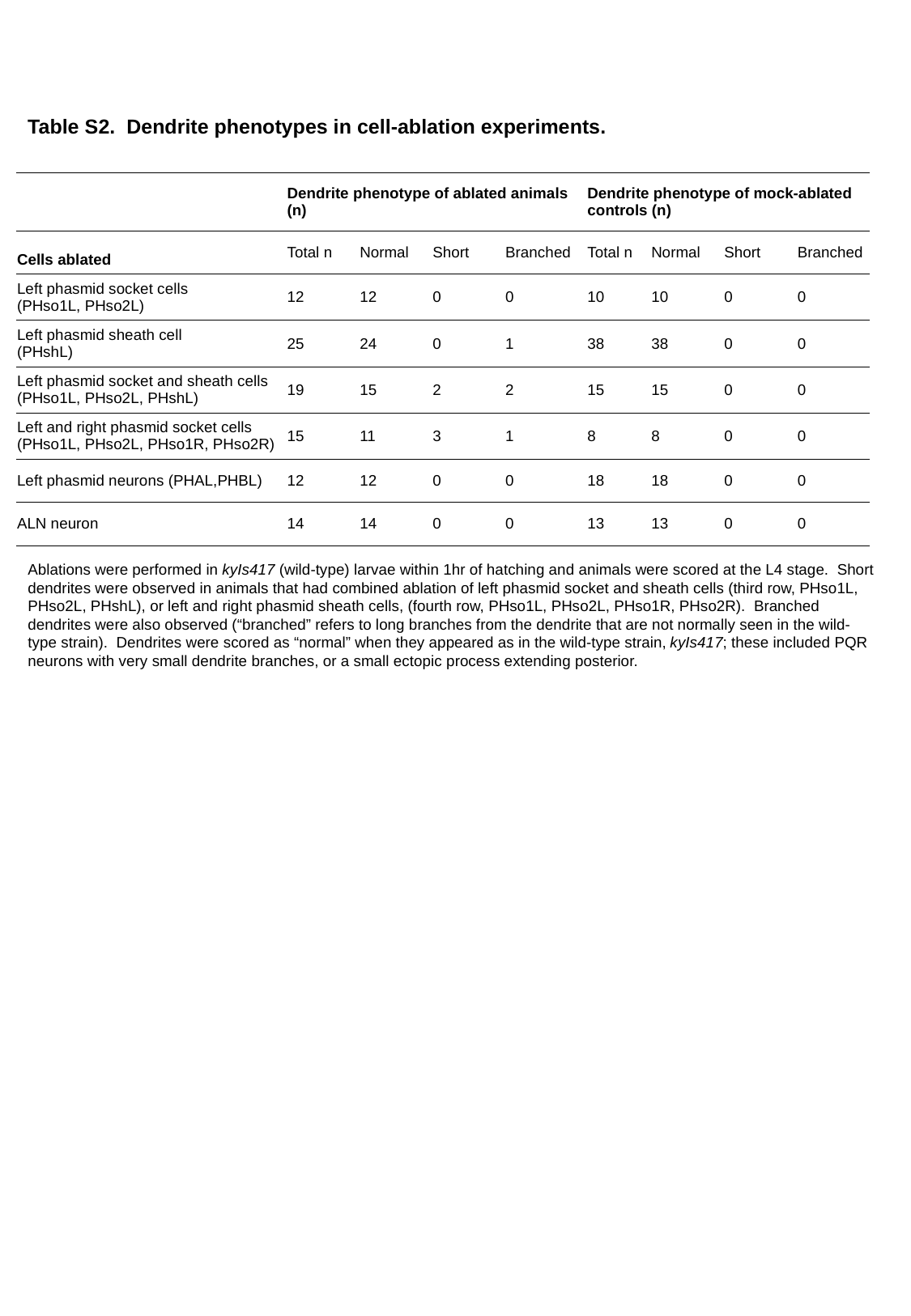

Table S2. Dendrite phenotypes in cell-ablation experiments.
| | Dendrite phenotype of ablated animals (n) | | | | Dendrite phenotype of mock-ablated controls (n) | | | |
| --- | --- | --- | --- | --- | --- | --- | --- | --- |
| Cells ablated | Total n | Normal | Short | Branched | Total n | Normal | Short | Branched |
| Left phasmid socket cells (PHso1L, PHso2L) | 12 | 12 | 0 | 0 | 10 | 10 | 0 | 0 |
| Left phasmid sheath cell (PHshL) | 25 | 24 | 0 | 1 | 38 | 38 | 0 | 0 |
| Left phasmid socket and sheath cells (PHso1L, PHso2L, PHshL) | 19 | 15 | 2 | 2 | 15 | 15 | 0 | 0 |
| Left and right phasmid socket cells (PHso1L, PHso2L, PHso1R, PHso2R) | 15 | 11 | 3 | 1 | 8 | 8 | 0 | 0 |
| Left phasmid neurons (PHAL,PHBL) | 12 | 12 | 0 | 0 | 18 | 18 | 0 | 0 |
| ALN neuron | 14 | 14 | 0 | 0 | 13 | 13 | 0 | 0 |
Ablations were performed in kyIs417 (wild-type) larvae within 1hr of hatching and animals were scored at the L4 stage. Short dendrites were observed in animals that had combined ablation of left phasmid socket and sheath cells (third row, PHso1L, PHso2L, PHshL), or left and right phasmid sheath cells, (fourth row, PHso1L, PHso2L, PHso1R, PHso2R). Branched dendrites were also observed (“branched” refers to long branches from the dendrite that are not normally seen in the wild-type strain). Dendrites were scored as “normal” when they appeared as in the wild-type strain, kyIs417; these included PQR neurons with very small dendrite branches, or a small ectopic process extending posterior.
