# Supplementary material for: LIN-44/Wnt Directs Dendrite Outgrowth through LIN-17/Frizzled in C. elegans Neurons
Source: PLoS Biol. 2011 Sep 20;9(9):e1001157. doi: 10.1371/journal.pbio.1001157 (PMC3176756; doi:10.1371/journal.pbio.1001157)
Supplement: Table S3 — PQR dendrite defects in single, double, and triple mutants. (PPT) [file pbio.1001157.s010.ppt]

## Slide 1
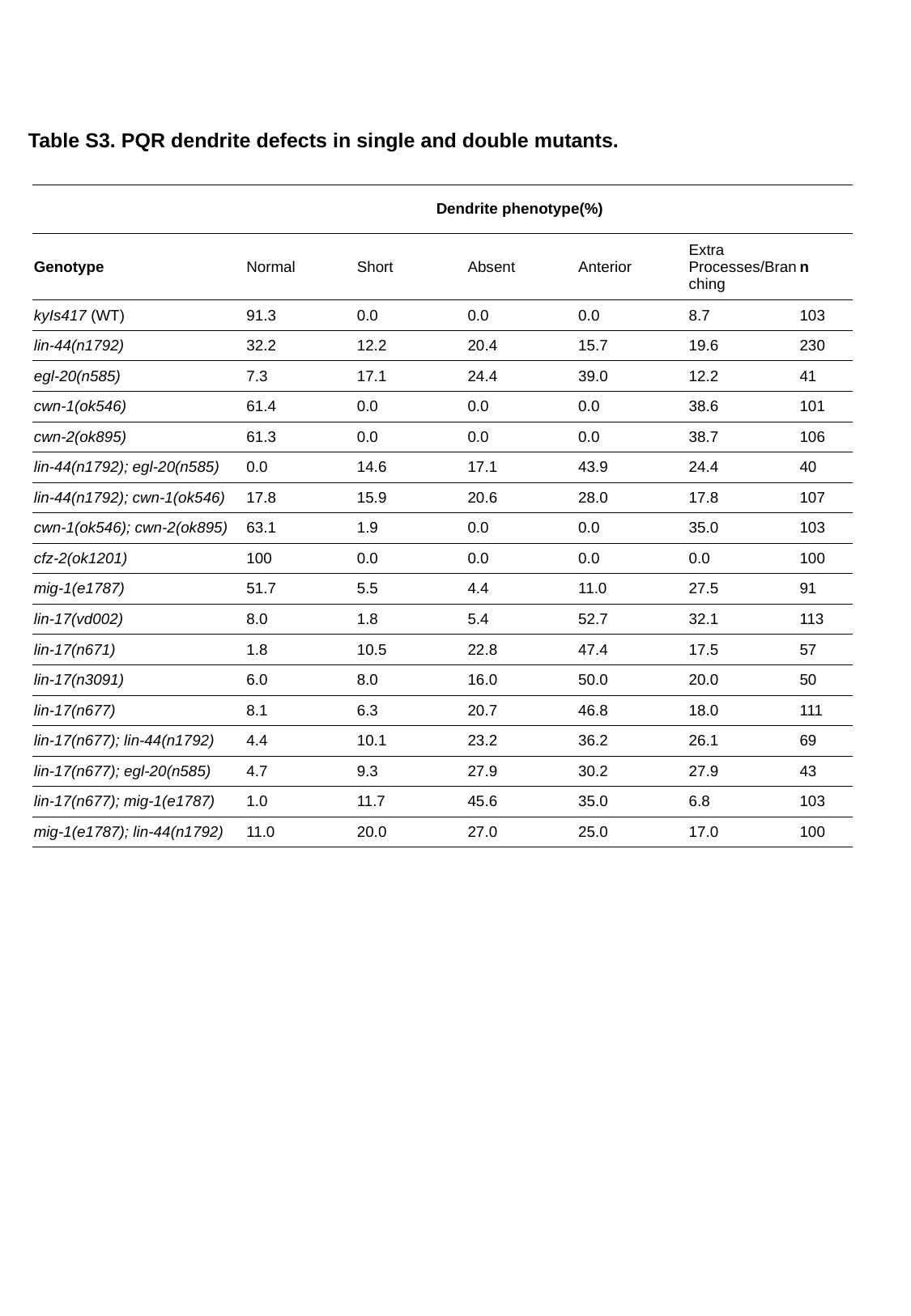

Table S3. PQR dendrite defects in single and double mutants.
| | Dendrite phenotype(%) | | | | | |
| --- | --- | --- | --- | --- | --- | --- |
| Genotype | Normal | Short | Absent | Anterior | Extra Processes/Branching | n |
| kyIs417 (WT) | 91.3 | 0.0 | 0.0 | 0.0 | 8.7 | 103 |
| lin-44(n1792) | 32.2 | 12.2 | 20.4 | 15.7 | 19.6 | 230 |
| egl-20(n585) | 7.3 | 17.1 | 24.4 | 39.0 | 12.2 | 41 |
| cwn-1(ok546) | 61.4 | 0.0 | 0.0 | 0.0 | 38.6 | 101 |
| cwn-2(ok895) | 61.3 | 0.0 | 0.0 | 0.0 | 38.7 | 106 |
| lin-44(n1792); egl-20(n585) | 0.0 | 14.6 | 17.1 | 43.9 | 24.4 | 40 |
| lin-44(n1792); cwn-1(ok546) | 17.8 | 15.9 | 20.6 | 28.0 | 17.8 | 107 |
| cwn-1(ok546); cwn-2(ok895) | 63.1 | 1.9 | 0.0 | 0.0 | 35.0 | 103 |
| cfz-2(ok1201) | 100 | 0.0 | 0.0 | 0.0 | 0.0 | 100 |
| mig-1(e1787) | 51.7 | 5.5 | 4.4 | 11.0 | 27.5 | 91 |
| lin-17(vd002) | 8.0 | 1.8 | 5.4 | 52.7 | 32.1 | 113 |
| lin-17(n671) | 1.8 | 10.5 | 22.8 | 47.4 | 17.5 | 57 |
| lin-17(n3091) | 6.0 | 8.0 | 16.0 | 50.0 | 20.0 | 50 |
| lin-17(n677) | 8.1 | 6.3 | 20.7 | 46.8 | 18.0 | 111 |
| lin-17(n677); lin-44(n1792) | 4.4 | 10.1 | 23.2 | 36.2 | 26.1 | 69 |
| lin-17(n677); egl-20(n585) | 4.7 | 9.3 | 27.9 | 30.2 | 27.9 | 43 |
| lin-17(n677); mig-1(e1787) | 1.0 | 11.7 | 45.6 | 35.0 | 6.8 | 103 |
| mig-1(e1787); lin-44(n1792) | 11.0 | 20.0 | 27.0 | 25.0 | 17.0 | 100 |
